# Efficacy and Safety of ACURATE neo2 in Valve-in-Valve TAVI: A Prospective Single-Center Study

**DOI:** 10.3390/jcm14134677

**Published:** 2025-07-02

**Authors:** Georgios E. Papadopoulos, Ilias Ninios, Sotirios Evangelou, Andreas Ioannidis, Athinodoros Nikitopoulos, George Giannakoulas, Vlasis Ninios

**Affiliations:** 1 Cardiology Department, Interbalkan Medical Center, 55535 Thessaloniki, Greece; georgios.e.papadopoulos@gmail.com (G.E.P.); iliasninios@gmail.com (I.N.); evangelousotirios@yahoo.com (S.E.); ioannides.andreas69@gmail.com (A.I.); dranikitopoulos@hotmail.com (A.N.); 2 Pulmonary Hypertension and Congenital Heart Disease Unit, AHEPA University General Hospital, 54636 Thessaloniki, Greece; g.giannakoulas@gmail.com

**Keywords:** transcatheter aortic valve implantation, valve-in-valve, bioprosthetic valve failure, ACURATE neo2, interventional cardiology, structural heart disease

## Abstract

**Background/Objectives**: Valve-in-valve (ViV) transcatheter aortic valve implantation (TAVI) is a key approach for treating degenerated surgical bioprosthetic valves. The ACURATE neo2 valve, with its advanced sealing technology and optimized coronary access, represents a promising solution for the challenges of ViV TAVI. This study evaluates the procedural and 30-day and 1-year follow-up outcomes of the ACURATE neo2 valve in ViV TAVI. **Methods**: This single-center, single-operator prospective study included patients with symptomatic bioprosthetic valve dysfunction, classified in New York Heart Association (NYHA) class III or IV, who underwent ViV TAVI with ACURATE neo2 at our center between July 2022 and February 2024. Outcomes were assessed using VARC-3 criteria. **Results**: Fifty-five patients (51% females, median (IQR) age 76 (8) years) were included. The technical success rate was 98.2%. No patients experienced in-hospital mortality, stroke, MI, bleeding, vascular complications, renal failure, or new pacemaker implantation. Three patients (5.5%) underwent elective chimney stenting for coronary protection. The postprocedural mean aortic gradient was 6.7 ± 1 mmHg, with a mean aortic valve area (AVA) of 2.0 ± 0.1 cm^2^. Over a median follow-up period of 1.2 years, no deaths (0%) were observed, heart failure hospitalization rate was 3.6%, and NYHA class improved to ≤II in 100% of patients. **Conclusions**: ACURATE neo2 demonstrated excellent technical success, sustained hemodynamic performance, and significant clinical improvement in ViV TAVI. The absence of major adverse events reinforces its safety, efficacy, and durability as a treatment for degenerated surgical bioprostheses.

## 1. Introduction

Surgical bioprosthetic valves are commonly selected for SAVR because they provide satisfactory hemodynamics and eliminate the need for chronic anticoagulation [1]. Their use has increased significantly, now accounting for approximately 80% of SAVR procedures [2]. However, bioprosthetic valves are inherently limited by structural valve degeneration (SVD), leading to progressive dysfunction over time. When bioprosthetic valve failure occurs, redo SAVR remains an option but is associated with substantial procedural risk, with reported operative mortality rates ranging from 2% to 7%, escalating to as high as 30% in elderly and high-risk patients [3]. Consequently, valve-in-valve (ViV) transcatheter aortic valve implantation (TAVI) has emerged as the preferred alternative for such patients, offering a minimally invasive approach with favorable early and mid-term outcomes [4,5,6].

Despite the procedural advantages of ViV TAVI, it poses specific technical and clinical challenges that must be considered. These include an increased risk of coronary obstruction, elevated postprocedural transvalvular gradients, permanent pacemaker implantation (PPI), and patient–prosthesis mismatch (PPM). The selection of the most appropriate transcatheter heart valve (THV) is therefore critical in optimizing both procedural success and long-term clinical outcomes.

**Figure 1:**
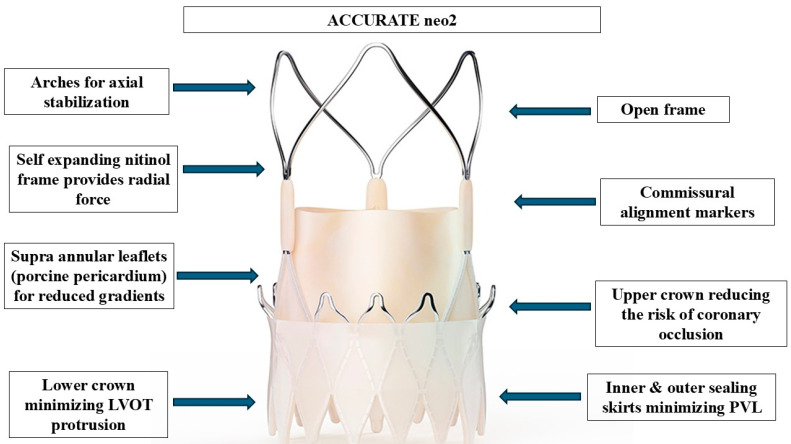
Structural design and functional features of the ACURATE neo2 transcatheter aortic valve. This self-expanding, supra-annular valve system is constructed from a nitinol frame that provides radial force and conforms to the native annulus. The valve incorporates supra-annular porcine pericardial leaflets, which optimize effective orifice area and reduce transvalvular gradients—particularly important in valve-in-valve (ViV) applications. The arches at the top of the frame serve to stabilize the valve axially during deployment and positioning. The open-cell frame architecture allows for enhanced visibility and improved coronary access post-implantation, while the commissural alignment markers facilitate optimal rotational orientation. The upper crown aids in anchoring while minimizing the risk of coronary obstruction, and the lower crown reduces left ventricular outflow tract (LVOT) protrusion. Both inner and outer pericardial sealing skirts contribute to minimizing paravalvular leak (PVL), a common concern in ViV TAVI procedures. Together, these features enable precise deployment, excellent hemodynamics, and high procedural safety in the context of treating degenerated surgical bioprostheses. Abbreviations: LVOT: left ventricular outflow tract; PVL: paravalvular leak.

The primary differences between self-expanding (SE) and balloon-expandable (BE) valves in the ViV setting pertain to their hemodynamic performance and risk profiles. SE valves, due to their supra-annular design, generally achieve a larger post-implantation aortic valve area (AVA) and lower incidence of PPM, making them advantageous in cases where elevated gradients are a concern; however, this comes at the cost of an increased risk of PPI and coronary obstruction, particularly in patients with low coronary ostia or externally mounted bioprosthetic leaflets [7,8]. While the most widely used SE valve for ViV TAVI is Evolut™ (Medtronic, Minneapolis, MN, USA), ACURATE neo2 (Boston Scientific, Marlborough, MA, USA) offers design modifications that may mitigate some of the risks associated with self-expanding THVs while introducing potential advantages over Evolut. Specifically, ACURATE neo2 retains the supra-annular design while incorporating an open-cell frame structure, which facilitates commissural alignment and preserves coronary access, thereby reducing the risk of coronary obstruction. Additionally, its stable positioning and simplified deployment mechanism makes it user-friendly, along with a lower incidence of conduction disturbances and PPI, an important advantage over other SE valves. The main structural features of the ACURATE neo2 valve are summarized in Figure 1.

While the performance of the ACURATE neo2 valve in native aortic stenosis has been well documented [9,10], data on its use in ViV TAVI remain limited. This study presents a single-center, single-operator experience evaluating the procedural success, hemodynamic performance, and 30-day and 1-year follow-up outcomes of the ACURATE neo2 valve in ViV TAVI, providing valuable insights into the real-world feasibility and safety of this approach.

## 2. Materials and Methods

This prospective, observational study was conducted at a single high-volume structural heart center and led by a single operator. It includes all consecutive patients who underwent transfemoral valve-in-valve (ViV) transcatheter aortic valve implantation (TAVI) using the ACURATE neo2 system between July 2022 and February 2024. Data were extracted from institutional medical records. The study protocol was approved by the Institutional Review Board in accordance with the ethical principles outlined in the Declaration of Helsinki.

### 2.1. Patient Selection

Eligible participants were those with symptomatic dysfunction of previously implanted surgical bioprosthetic aortic valves, categorized as New York Heart Association (NYHA) functional class III or IV. All ViV TAVI procedures were performed in the hybrid operating suite of the European Interbalkan Medical Center. The decision for transcatheter intervention was made by a multidisciplinary heart team, following the recommendations of the 2021 ESC/EACTS guidelines for valvular heart disease [11].

### 2.2. ViV TAVI Procedure

The transfemoral ViV TAVI procedure was performed under general anesthesia in the Hybrid Operating Room of the Interbalkan Medical Center, utilizing the ACURATE neo2 (Boston Scientific, Marlborough, MA, USA), by a single operator. Femoral artery access was obtained through a surgical cut-down technique by the same vascular surgeon.

Crossing of the bioprosthetic valve was facilitated by a straight 0.035” wire through an Amplatz Left 1 or a pigtail catheter. Balloon predilation was performed in most cases using a balloon sized according to the true internal diameter of the surgical bioprosthesis. In patients with small prostheses (true ID < 21 mm), high-pressure balloon fracture was considered when applicable, using a non-compliant Atlas balloon (Bard Peripheral Vascular, Tempe, AZ, USA) prior to ACURATE neo2 deployment in order to optimize valve expansion and reduce residual gradients and to avoid high-pressure trauma to the ACURATE neo2 leaflets.

ACURATE neo2 implantation followed a standardized top-down deployment approach, ensuring that the valve marker was aligned with the bioprosthetic valve annulus. Postdilation was selectively performed in cases where suboptimal valve expansion, elevated residual gradients, or significant paravalvular regurgitation were observed. Following implantation, a pigtail catheter was positioned in the left ventricle to assess residual gradients and perform hemodynamic measurements, including diastolic pressures in the aorta and left ventricle.

In patients with low coronary artery takeoff, preemptive coronary protection was performed using guide catheters and coronary angioplasty wires. Elective chimney stenting was undertaken to preserve coronary flow, when necessary.

### 2.3. Definition of the Variables

Baseline data collected for all patients included demographic information (age, sex, body mass index [BMI]) and clinical risk factors such as estimated glomerular filtration rate (eGFR) and EuroSCORE II. Cardiopulmonary comorbidities were recorded, including prior heart failure admissions, atrial fibrillation (AF), previous myocardial infarction (MI), diabetes, hypertension, dyslipidemia, and coronary artery disease (CAD)—with or without a history of percutaneous coronary intervention (PCI) or coronary artery bypass grafting (CABG). Additional variables included chronic obstructive pulmonary disease (COPD), prior stroke, the presence of a permanent pacemaker, and New York Heart Association (NYHA) functional class. Detailed information on the failed surgical bioprosthesis was also documented, including true internal diameter (ID), time to valve degeneration, underlying mechanism of failure (stenosis, regurgitation, or mixed), and the original valve manufacturer. Baseline transthoracic echocardiography (TTE) was performed in all patients to evaluate left ventricular ejection fraction (LVEF), mean transvalvular aortic gradient, aortic valve area (AVA), and the presence and severity of aortic regurgitation (AR), which was categorized as trivial/mild, moderate, or severe. All TTE assessments were performed by a single experienced echocardiographer to ensure consistency. Procedural data included predilation and postdilation status, use of the SENTINEL cerebral protection system, total procedural duration, and commissural alignment.

### 2.4. In-Hospital Outcomes

Clinical outcomes during the index hospitalization were adjudicated using the Valve Academic Research Consortium-3 (VARC-3) definitions [12]. Key endpoints included technical success, in-hospital mortality, myocardial infarction, periprocedural PCI, stroke, bleeding events (of any severity), acute kidney injury (AKI), conversion to open surgery, vascular access complications, and the need for permanent pacemaker implantation. Postprocedural echocardiographic parameters were also assessed before discharge.

### 2.5. The 30-Day and 1-Year Follow-Up Outcomes

The 30-day and 1-year follow-up clinical and echocardiographic outcomes were assessed at 30 days and 1 year postprocedure. Clinical follow-up included evaluation of functional status (NYHA class), heart failure hospitalizations, all-cause mortality, myocardial infarction (MI), and stroke.

Echocardiographic follow-up was conducted at 30 days and 1 year using transthoracic echocardiography (TTE) to assess mean aortic valve gradient, aortic valve area (AVA), and aortic regurgitation (AR). AR severity was classified as trivial/mild, moderate, or severe. All echocardiographic evaluations were performed by the same examiner.

Device-related complications, including bioprosthetic valve dysfunction (stenosis, regurgitation, or combined), valve thrombosis, endocarditis, and structural valve deterioration, were also recorded. Follow-up data were collected through hospital visits, outpatient clinic records, and telephone interviews with patients or referring physicians.

### 2.6. Statistical Analysis

Continuous variables were assessed for normality using a combination of visual inspection (histograms and distribution plots), comparison of mean and median values, and the Kolmogorov–Smirnov test. Variables with normal distribution are expressed as mean ± standard deviation (SD), whereas non-normally distributed data are reported as median with interquartile range (IQR). Categorical variables are summarized using absolute and relative frequencies (n/N, %). To evaluate changes in continuous variables over multiple time points (e.g., baseline, postprocedure, 30-day, and 1-year follow-up), repeated measures analysis of variance (ANOVA) was used for normally distributed data, while the Friedman test was applied to non-parametric data. Post hoc pairwise comparisons were performed using paired *t*-tests or the Wilcoxon signed-rank test, as appropriate. Bonferroni correction was applied to account for multiple testing. A two-tailed *p*-value of <0.05 was considered indicative of statistical significance. All analyses were conducted using RStudio (version 2023.03.0+386).

## 3. Results

A total of 55 patients (51% females) underwent transfemoral ViV TAVI with a median age of 76 years (IQR: 8), at baseline assessment. Table 1 provides a detailed summary of the patients’ baseline demographic, clinical, and echocardiographic features. This is a high-surgical-risk cohort with a mean Euroscore II of 7.66 ± 0.9% and prevalent cardiopulmonary comorbidities, including hypertension (55%), dyslipidemia (67%), diabetes (27%), COPD (31%), and CAD (45%) or previous PCI (21%), CABG (24%), or MI (13%), while three (5%) patients had had a prior stroke and 11 (20%) had a permanent pacemaker. AF was present in 33% of patients. The failed surgical bioprostheses had a mean true internal diameter (ID) of 22 ± 3 mm, with a mean time to failure of 10.0 ± 4.1 years. The most common failure mechanism was stenosis (51%), followed by mixed degeneration (27%) and regurgitation (22%). The most frequently failed bioprosthetic valve was TRIFECTA (49%), followed by MAGNA EASE, MITROFLOW, and MOSAIC (each 14.5%). Preprocedural echocardiography showed a mean LVEF of 50 ± 11%, mean aortic gradient of 38 ± 11 mmHg, and AVA of 0.8 ± 0.5 cm^2^. Aortic regurgitation was trivial/mild in 31%, moderate in 40%, and severe in 39% of cases.

The technical success rate was 98.2%, with one patient requiring the implantation of a second ACURATE neo2 valve during the index procedure due to valve migration of the first postdilation and severe paravalvular leak (PVL) (Table 2). Predilation was performed in 87% (in five [9%] cases the surgical valve was fractured), postdilation in 66%, and the SENTINEL cerebral protection system was used in all patients. The mean procedural time was 32 ± 5 min. There were no occurrences of stroke, bleeding, vascular complications, AKI, conversion to surgery, new pacemaker implantation, or in-hospital mortality. Additionally, none of the patients experienced myocardial infarction; however, three patients (5.5%) underwent elective percutaneous coronary intervention with chimney stenting—two (3.6%) in the left main and one (1.8%) in both the left main and right coronary artery—due to low coronary takeoff and potential obstruction. The postprocedural mean aortic gradient was 6.7 ± 1 mmHg, with a mean aortic valve area (AVA) of 2 ± 0.1 cm^2^. No cases of aortic regurgitation greater than trivial were observed.

### The 30-Day and 1-Year Follow-Up Outcomes

**Figure 2:**
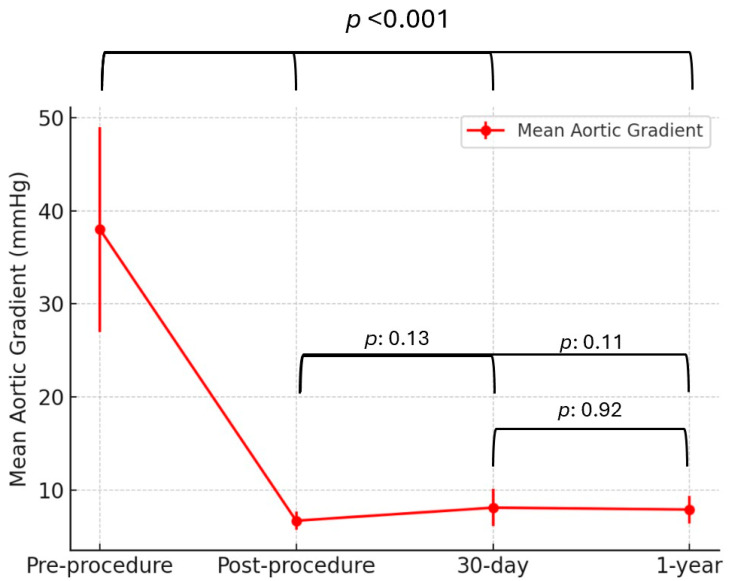
Mean aortic gradient over time following ViV TAVI with the ACURATE neo2 valve. Data are presented as mean ± standard deviation. Mean aortic gradient significantly decreased from preprocedure to postprocedure and remained stable at 30-day and 1-year follow-up.

Over a median follow-up period of 1.2 years, no deaths, strokes, or MIs (0%) were observed, and the rate of heart failure-related hospitalizations was 3.6%. The mean aortic gradient demonstrated a significant reduction following ViV TAVI, decreasing from 38 ± 11 mmHg preprocedure to 6.7 ± 1 mmHg postprocedure (*p* < 0.001). This reduction remained stable with 8.1 ± 2 mmHg at 30 days and 7.9 ± 1.5 mmHg at 1 year, with no significant differences between postprocedural, 30-day, and 1-year gradients (*p*: 0.13, *p*: 0.11, *p*: 0.92, Figure 2).

**Figure 3:**
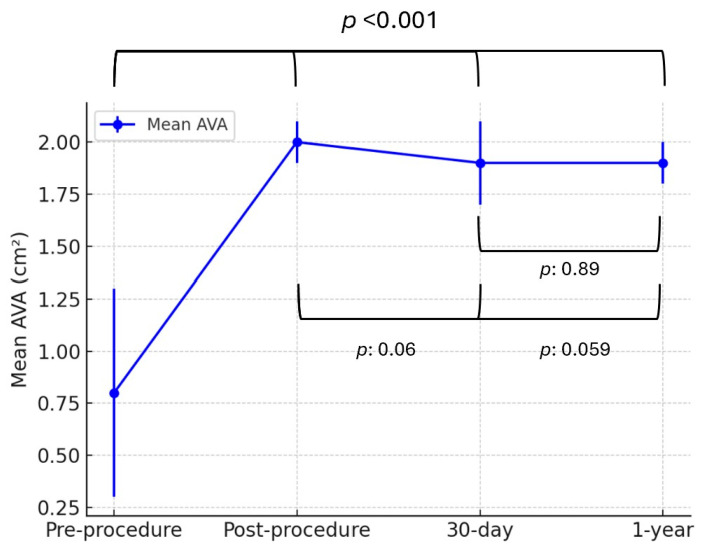
Mean aortic valve area (AVA) over time following ViV TAVI with the ACURATE neo2 valve. Data are presented as mean ± standard deviation. AVA significantly increased postprocedure and remained stable over time. Abbreviations: AVA: aortic valve area.

Similarly, the mean aortic valve area (AVA) significantly improved from 0.8 ± 0.5 cm^2^ preprocedure to 2.0 ± 0.1 cm^2^ postprocedure (*p* < 0.001). AVA remained stable at follow-up, measuring 1.9 ± 0.2 cm^2^ at 30 days and 1.9 ± 0.1 cm^2^ at 1 year, with no significant differences between postprocedural, 30-day, and 1-year measurements (*p*: 0.89, *p*: 0.06, *p*: 0.059, Figure 3).

**Figure 4:**
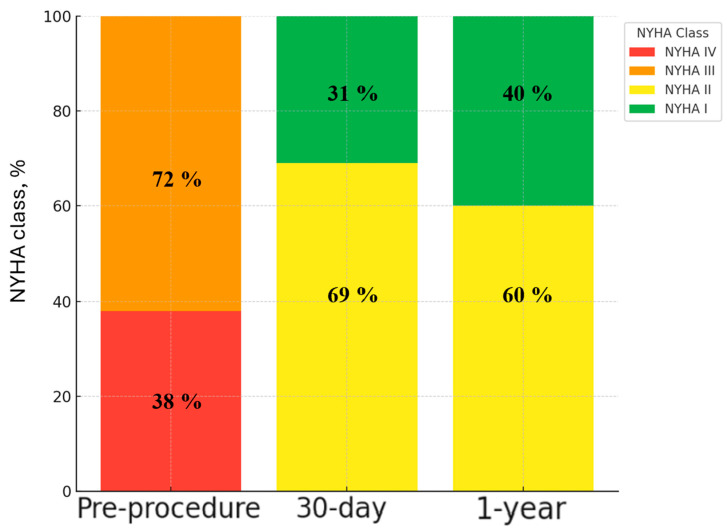
Mean New York Heart Association (NYHA) distribution over time following ViV TAVI with the ACURATE neo2 valve. All patients achieved NYHA class ≤ II at 30-day and 1-year follow-up. Abbreviations: NYHA: New York Heart Association.

Significant functional improvement was observed following ViV TAVI, as reflected in the distribution of NYHA class over time (Figure 4). Preprocedurally, 72% of patients were in NYHA class III and 38% in NYHA class IV. At 30-day follow-up, there was a marked shift towards improved functional status, with 31% of patients in NYHA class I and 69% in NYHA class II, and no patients remaining in class III or IV. This trend continued at 1-year follow-up, with further functional improvement, as 40% of patients were classified as NYHA I and 60% as NYHA II.

## 4. Discussion

This study represents a significant contribution to the growing body of evidence on the use of the ACURATE neo2 valve in the ViV TAVI setting. While the AVENGER registry [13] has provided valuable comparative data between ACURATE neo2 and Evolut in ViV TAVI, our study offers a distinct perspective by evaluating ACURATE neo2 in a dedicated single-center, single-operator setting. This controlled approach minimizes inter-operator variability and procedural heterogeneity, thereby allowing for a more precise assessment of the valve’s technical performance, safety profile, and 30-day and 1-year follow-up hemodynamic outcomes.

Our findings confirm that ViV TAVI with ACURATE neo2 is associated with high technical success and excellent hemodynamic performance, as reflected by a substantial and sustained reduction in mean transvalvular gradients and a significant improvement in AVA. The supra-annular leaflet design, a defining characteristic of self-expanding THVs [14], likely contributed to these favorable hemodynamic outcomes by allowing for larger effective orifice areas (EOAs) and mitigating the risk of PPM, which remains a critical concern in the ViV setting, particularly in smaller surgical bioprostheses [15], which in the VIVID (Valve-in-Valve International Data) registry were associated with increased mortality after ViV procedure [16].

A key distinguishing feature of our study is the high rate of predilation, which is not generally recommended in ViV TAVI due to concerns about acute severe aortic insufficiency and hemodynamic instability [17]. Nevertheless, in our cohort, this strategy was employed systematically, after the very first few cases, to optimize valve expansion and mitigate residual gradients. Additionally, in five cases, a deliberate strategy of bioprosthetic valve fracture was performed to enhance post-implantation valve function. This approach is increasingly recognized as a valuable adjunct in cases with small true internal diameters, as it may facilitate a larger final valve area and reduce postprocedural gradients [18,19,20,21,22,23].

Importantly, in one case, migration of the initially implanted ACURATE neo2 necessitated the implantation of a second transcatheter valve, resulting in a successful valve-in-valve-in-valve (ViV-in-ViV) procedure. This finding underscores the feasibility of ViV-in-ViV as a bailout strategy in cases of valve migration or suboptimal positioning, particularly in bioprosthetic valves with low internal diameters or incomplete frame expansion. While ViV-in-ViV is not yet widely studied, its potential to restore optimal hemodynamics without requiring surgical conversion warrants further investigation.

From a safety standpoint, the absence of in-hospital mortality, myocardial infarction, major vascular complications, or new pacemaker implantation underscores the procedural feasibility of ACURATE neo2 in ViV TAVI. In particular, the absence of vascular complications (0%) in our cohort is noteworthy when contrasted with earlier experiences using the first-generation ACURATE neo valve. In the multicenter registry by Kim et al., vascular complications were reported in 6.9% of patients undergoing TAVI with the ACURATE neo system [24]. This marked difference may be attributed to both the design improvements introduced in the ACURATE neo2 and the procedural consistency afforded by a single-operator, single-center approach. The lower profile and enhanced delivery system of the ACURATE neo2 likely contributed to a reduction in vascular trauma. Moreover, the systematic use of surgical femoral access in all cases may have further minimized access-site complications by ensuring optimal arterial control. This technique provides direct arterial visualization and superior hemostatic control compared to percutaneous methods. Contrary to the perception that this approach prolongs recovery, our previously published propensity-matched analysis comparing surgical and percutaneous femoral access in transfemoral TAVI found that surgical access was associated with significantly lower vascular and bleeding complications, no in-hospital mortality, and even shorter hospitalization durations [25]. These findings support the clinical and logistical feasibility of this approach.

Another notable safety finding in this study was the absence of stroke (0%), which may be attributed to the routine use of the SENTINEL cerebral protection system in all patients. The SENTINEL device is designed to capture embolic debris that may arise during ViV TAVI, particularly in cases requiring predilation, bioprosthetic valve fracture, or postdilation—maneuvers that increase the risk of embolization. The complete prevention of clinically evident strokes in our cohort underscores the potential role of systematic SENTINEL use in reducing neurological complications, suggesting that embolic protection may be particularly beneficial in ViV TAVI settings where pre-existing bioprosthetic calcification and debris fragmentation may pose an increased risk of embolization [26]. These findings align with prior studies that have suggested a protective effect of embolic protection devices, though larger trials are needed to confirm their impact on neurocognitive outcomes [27,28,29,30]. These findings are consistent with earlier observational studies suggesting the benefit of embolic protection devices [27,28,29,30]. However, recent large, randomized trials, including the PROTECTED TAVR [31] and BHF PROTECT-TAVI [32], have reported neutral or negative results regarding the impact of routine cerebral embolic protection on stroke prevention in the broader TAVI population. Importantly, these trials predominantly enrolled patients undergoing TAVI for native valve disease and may not fully reflect the risk profile of ViV TAVI procedures. Therefore, while routine use of cerebral embolic protection may not be universally warranted, our data support its selective application in high-risk scenarios such as ViV TAVI, particularly when performed with adjunctive high-risk maneuvers.

A particularly significant safety observation in our study was the complete absence of bleeding complications, including minor (Type 1) events as defined by VARC-3 criteria. In our cohort, this outcome likely reflects a combination of factors, including a standardized antithrombotic protocol and, more importantly, the routine use of surgical femoral artery access via cut-down. All patients received dual antiplatelet therapy (aspirin 100 mg and clopidogrel 75 mg daily) for 6 months postprocedure, followed by long-term aspirin monotherapy. For patients with chronic atrial fibrillation, oral anticoagulation was continued per baseline indication. Intraprocedural anticoagulation consisted of unfractionated heparin targeting an ACT > 250 s. However, the most critical contributor to the absence of vascular and bleeding events was the consistent use of surgical cut-down for femoral access, performed by an experienced vascular team.

The importance of careful preprocedural planning is further highlighted by the requirement for PCI with chimney stenting in 5.5% of patients, owing to the risk of coronary obstruction, particularly in patients with low coronary takeoff or externally mounted leaflets. The open-cell frame design of ACURATE neo2 may provide an advantage in such cases, allowing for improved coronary access and commissural alignment, thereby reducing the complexity of bailout coronary interventions [14].

The durability of hemodynamic improvements was evident at both 30-day and 1-year follow-up, with stable gradients and AVA, as well as significant functional improvement reflected in NYHA class. These findings suggest that ACURATE neo2 offers sustained clinical benefits beyond the periprocedural period. Given the increasing reliance on ViV TAVI as an alternative to redo surgery, long-term durability data will be essential to establish the role of ACURATE neo2 in this patient population.

While our study provides important insights, certain limitations should be acknowledged. The single-center, single-operator nature of the study, while ensuring procedural consistency, may limit external validity. Additionally, the absence of a comparator group precludes direct assessment of ACURATE neo2 relative to other THVs in the ViV setting. It is important to note that the results reported herein reflect a single-center experience with procedures performed by a high-volume, expert operator. While this uniformity enhances internal validity and minimizes procedural variability, it may limit generalizability to lower-volume centers or operators with different experience levels. Therefore, caution should be exercised when extrapolating these findings to broader clinical practice. Larger multicenter studies are needed to confirm these outcomes across diverse real-world settings. Future multicenter studies, as well as head-to-head comparisons, will be necessary to further delineate the optimal THV choice in this context.

## 5. Conclusions

This study reinforces the feasibility, safety, and hemodynamic efficacy of ACURATE neo2 in ViV TAVI, with excellent 30-day and 1-year follow-up outcomes. The supra-annular design and open-cell frame structure contribute to favorable hemodynamics, while the low incidence of conduction disturbances and preserved coronary access are important procedural advantages. In the absence of large-scale data, our findings provide valuable real-world insights into the performance of ACURATE neo2 in a dedicated ViV TAVI setting. Further studies with long-term follow-up and comparative analyses are warranted to refine patient and device selection for optimal outcomes in this challenging patient population.

## Figures and Tables

**Table 1:** Summary of baseline patient characteristics.

Characteristic	Overall, N = 55 ^1^
Age, years	78 (6)
Female sex	28/55 (51)
BMI, kg/m^2^	25.5 (1)
Euroscore II, %	7.66 ± 0.9
NYHA class IV	21/55 (38)
Hypertension	30/55 (55)
Dyslipidemia	37/55 (67)
Diabetes	15/55 (27)
CAD	25/55 (45)
Previous PCI	12/55 (22)
Previous CABG	13/55 (24)
Previous MI	7/55 (13)
Stroke	3/55 (5)
Pacemaker	11/55 (20)
AF	18/55 (33)
COPD	17/55 (31)
eGFR, mL/min/1.73 m^2^	56 ± 17
True ID, mm	22 ± 3
Time to failure, years	10.0 ± 4.1
Degeneration mechanism	
Stenosis	28/55 (51)
Regurgitation	12/55 (22)
Mixed	15/55 (27)
Surgical valve	
MAGNA EASE	8/55 (14.5)
MITROFLOW	8/55 (14.5)
MOSAIC	8/55 (14.5)
SORIN CROWN	2/55 (3.6)
St JUDE EPIC 23	2/55 (3.6)
TRIFECTA	27/55 (49)
Echocardiography	
LVEF, %	50 (15)
Mean aortic gradient, mmHg	38 ± 11
AVA, cm^2^	0.8 ± 0.5
AR	
Trivial/Mild	17/55 (31)
Moderate	22/55 (40)
Severe	16/55 (29)

^1^ Mean ± SD; Median (IQR); n/N (%). Note: Continuous data are presented as mean ± SD, while categorical data are summarized as absolute and relative frequencies (n/N, %). Abbreviations: BMI: body mass index; Euroscore II: European System for Cardiac Operative Risk Evaluation II; NYHA: New York Heart Association; CAD: coronary artery disease; PCI: percutaneous coronary intervention; CABG: coronary artery bypass graft surgery; MI: myocardial infarction; AF: atrial fibrillation; COPD: chronic obstructive pulmonary disease; eGFR: estimated glomerular filtration rate; LVEF: left ventricular ejection fraction; AVA: aortic valve area; AR: aortic regurgitation.

**Table 2:** Procedural results.

Characteristic	Overall, N = 55 ^1^
Technical success	54/55 (98.2)
Predilation	48/55 (87)
Surgical valve fracture	5/55 (9)
Postdilation	36/55 (65)
SENTINEL cerebral protection system	55/55 (100)
Procedural time, mins	32 ± 5
Commissural alignment	55/55 (100)
Stroke	0/55 (0)
Bleeding	0/55 (0)
Vascular complications	0/55 (0)
AKI	0/55 (0)
Conversion to surgery	0/55 (0)
Pacemaker implantation	0/55 (0)
In-hospital mortality	0/55 (0)
Myocardial infarction	0/55 (0)
Elective PCI—chimney stenting	3/55 (5.5)
LM	2/55 (3.6)
LM + RCA	1/55 (1.8)
Postprocedural mean aortic gradient, mmHg	6.7 ± 1
Mean AVA, cm^2^	2 ± 0.1
AR	0/55 (0)
Trace	55/55 (100)
Mild	0/55 (0)
Moderate	0/55 (0)
Severe	0/55 (0)
Hospital stay, days	3.2 ± 0.6
Home—discharged	55/55 (100)
30-day mortality	0/55 (0)

^1^ Mean ± SD; n/N (%). Note: Continuous variables are presented as mean value ± standard deviation (SD). Categorical variables are presented as n/N (%). Abbreviations: AKI: acute kidney injury; PCI: percutaneous coronary intervention; LM: left main; RCA: right coronary artery; AVA: aortic valve area; AR: aortic regurgitation.

## Data Availability

The data presented in this study are available on request from the corresponding author. The data are not publicly available due to ethical restrictions.

## Abbreviations

The following abbreviations are used in this manuscript:

AF	Atrial Fibrillation
AKI	Acute Kidney Injury
AR	Aortic Regurgitation
AVA	Aortic Valve Area
BE	Balloon-Expandable (valve)
BMI	Body Mass Index
CABG	Coronary Artery Bypass Graft Surgery
CAD	Coronary Artery Disease
COPD	Chronic Obstructive Pulmonary Disease
EACTS	European Association for Cardio-Thoracic Surgery
ECS	European Society of Cardiology
EOA	Effective Orifice Area
EuroSCORE II	European System for Cardiac Operative Risk Evaluation II
eGFR	Estimated Glomerular Filtration Rate
IQR	Interquartile Range
ID	True Internal Diameter
LM	Left Main (coronary artery)
LVEF	Left Ventricular Ejection Fraction
MI	Myocardial Infarction
NYHA	New York Heart Association (functional class)
PCI	Percutaneous Coronary Intervention
PPI	Permanent Pacemaker Implantation
PPM	Patient–Prosthesis Mismatch
PVL	Paravalvular Leak
RCA	Right Coronary Artery
SAVR	Surgical Aortic Valve Replacement
SE	Self-Expanding (valve)
SENTINEL	Sentinel Cerebral Protection System
SVD	Structural Valve Degeneration
TAVI	Transcatheter Aortic Valve Implantation
THV	Transcatheter Heart Valve
TTE	Transthoracic Echocardiography
VARC-3	Valve Academic Research Consortium-3
